# Right ventricular remodeling in athletes and crista supraventricularis pattern

**DOI:** 10.1002/clc.23383

**Published:** 2020-05-23

**Authors:** Manuel Martinez‐Sellés, Leonel Diaz‐Gonzalez, Alejandro Lucia

**Affiliations:** ^1^ Cardiology Department, Hospital Universitario Gregorio Marañón, CIBERCV Universidad Europea. Universidad Complutense Madrid Spain; ^2^ Cardiology Department CEMTRO Clinic Madrid Spain; ^3^ Faculty of Sport Sciences European University and Research Institute Hospital 12 de Octubre (‘imas12’) Madrid Spain

To the Editor,

We read with interest the excellent review article on right ventricular remodeling in athletes by Sanz‐de la Garza et al.^1^ Electrocardiographic (ECG) assessment is the first step in the proposed algorithm. In this regard, international criteria for a correct interpretation of athletes ECG were recently published.^2^ Yet, some issues remain. Notably, incomplete right bundle branch block is a prevalent ECG manifestation in athletes, being present in 10% to 30%.^3^ The common explanation for this phenomenon is the dilation of right ventricle after years of intense sports practice, which in turn delays intraventricular conduction through the right bundle branch block.^4^ Differences in the QRS duration criteria used to identify incomplete right bundle branch block (<120 vs >100 ms and <120 ms) further complicates this question, as a conduction delay seems unlikely when QRS < 100 ms.^5^ In the general population, coexistence of QRS width ≤100 ms and an RSR′ pattern in lead V1 is known as *crista supraventricularis* pattern, where *crista supraventricularis* is a right ventricular muscular ridge located between the tricuspid and pulmonic valve that is fully supplied by the Purkinje network and is one of the last structures to be depolarized.^6^ *Crista supraventricularis* pattern might result from posterior apex deviation, subpulmonic area delay, or late *crista supraventricularis* activation.^7^, ^8^ The latter is reflected by the presence of S waves in leads I, II, and III (ie, the so‐called “S1S2S3” pattern). The prevalence of *crista supraventricularis* pattern has not been studied in athletes. This is, however, an important question. Indeed, the mechanisms for *crista supraventricularis* pattern occurrence seem to differ from those causing incomplete right bundle branch block. The differentiation between these two ECG manifestations may prevent misdiagnosis of actual *crista supraventricularis* pattern as incomplete right bundle branch block allowing a better electrocardiographic characterization in athletes.
